# Thyroglossal duct pathology and mimics

**DOI:** 10.1186/s13244-019-0694-x

**Published:** 2019-02-06

**Authors:** Swapnil Patel, Alok A. Bhatt

**Affiliations:** Department of Imaging Sciences, University of Rochester Medical Center, 601 Elmwood Avenue, P.O. Box 648, Rochester, NY 14642 USA

**Keywords:** Thyroglossal duct, Ectopic thyroid, Neck mass, Cystic lesions

## Abstract

Congenital anterior neck masses comprise a rare group of lesions typically diagnosed in childhood. Most commonly, lesions are anomalies of the thyroglossal duct, namely the thyroglossal duct cyst, along with ectopic thyroid tissue. Although usually suspected based on clinical examination, imaging can confirm the diagnosis, assess the extent, and evaluate for associated complications. Imaging characteristics on ultrasound, CT, and MRI may at times be equivocal; differential considerations include branchial cleft cyst, dermoid/epidermoid, laryngocele, thymic cyst, lymphatic malformation, and metastatic disease. Thus, understanding of the embryologic course of thyroid development is crucial with recognition of critical landmarks such as the foramen cecum, hyoid bone, thyroid cartilage, and strap musculature to aid in the diagnosis of an anterior neck mass.

## Teaching points

- Thyroglossal duct cyst is the most common congenital neck mass.
- Critical anatomic landmarks of thyroglossal duct anomalies and ectopic thyroid tissue include the foramen cecum at the tongue base, hyoid bone, thyroid cartilage, and strap musculature.
- Important differentiating feature of thyroglossal duct cyst is close association to the posterior aspect of the hyoid bone.
- Suprahyoid thyroglossal duct cysts are usually midline, while infrahyoid thyroglossal duct cysts may be paramidline.
- Orthotopic thyroid tissue is absent in 70–80% of patients with lingual thyroid, and therefore, an important consideration in preoperative planning.

## Introduction

In the evaluation of anterior neck masses in children and young adults, thyroglossal duct anomalies are on top of the differential diagnosis. Subsequent diagnostic imaging with initial ultrasound examination followed by definitive CT and MR examinations assist in diagnosis and assessment of anatomical extent and complications, as well as pretreatment planning. In addition, knowledge of the course of the embryologic thyroid improves diagnostic accuracy. Critical anatomic landmarks of thyroglossal duct anomalies and ectopic thyroid tissue include the foramen cecum at the tongue base, hyoid bone, thyroid cartilage, and strap musculature.

The purpose of this article is to review the common and variant forms of thyroglossal duct anomalies. Anatomy of the embryologic descent of the thyroid gland will first be reviewed, followed by the imaging characteristics of thyroglossal duct cyst and ectopic thyroid tissue. Variant forms and complications of thyroglossal duct cysts will also be reviewed. To strengthen diagnostic accuracy of thyroglossal duct anomalies, similar appearing cystic anterior neck masses will be discussed with focus on key differentiating features.

### Anatomy and embryology

**Fig. 1:**
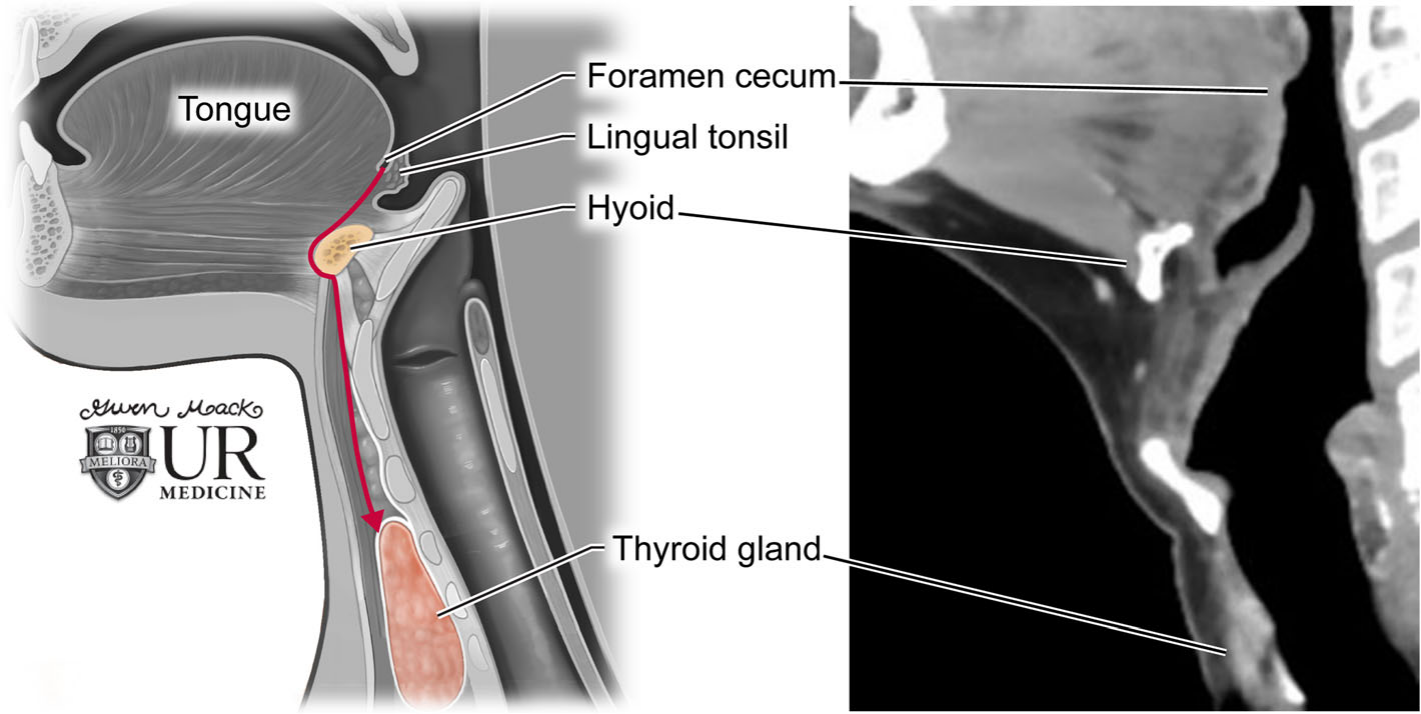
Thyroglossal duct anatomy. Illustration of the anatomic course of the thyroglossal duct during embryological development (left). Corresponding anatomy on a sagittal contrast-enhanced neck CT (right)

The thyroglossal duct is a transient epithelial lined midline channel serving as the path of descent of the thyroid primordium from the foramen cecum, located at the junction of the anterior two-thirds and posterior third of the tongue, down to the thyroid cartilage where definitive thyroid formation occurs [1]. The inferior portion of the duct may develop into the pyramidal lobe and the remainder involutes by the 10th week of gestation (Fig. 1).

### Role of imaging

Although benign anterior neck masses such as thyroglossal duct cysts are often diagnosed clinically, the clinical presentation of infected cysts, thyroglossal duct carcinoma, or other pathologic mimics may be indistinguishable, necessitating diagnostic imaging. In children or adults with low clinical suspicion for tumor, imaging evaluation may begin with ultrasound. However, if there are atypical sonographic features (i.e., solid component or abnormal vascularity) or high clinical suspicion for tumor, CT or MR imaging is recommended to document an orthotopic thyroid gland and to evaluate for and characterize the features and extent of neoplastic processes [2]. In selected cases, diffusion-weighted or dynamic contrast-enhanced MR imaging can be performed in evaluation of vascular malformations, abscess, or suspicious cervical lymph nodes [3].

### Thyroglossal duct cyst

If a portion of the thyroglossal duct persists, cystic lesions may arise following cycles of infection and/or inflammation as it is lined with secretory epithelium [4]. Cysts can form anywhere along the course of the duct; however, about 65% of cysts occur at the infrahyoid level [5]. The cyst is histologically composed of epithelial lining of squamous or pseudostratified ciliated columnar epithelium with or without ectopic thyroid gland tissue [6]. As secretions and debris accumulate, suprahyoid cysts can enlarge, push through the floor of the mouth and penetrate down into the anterior neck with resultant symptoms leading to clinical presentation. Thyroglossal duct cysts are the most common non-odontogenic cyst in the neck and most common pediatric cystic neck anomaly and are therefore important to recognize [7].

**Fig. 2:**
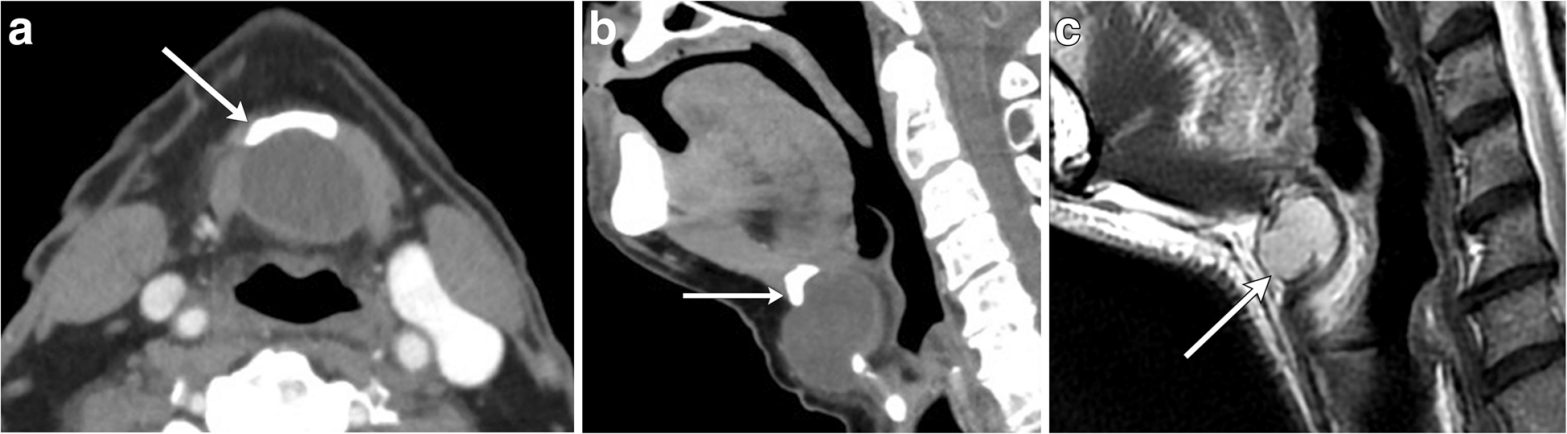
Midline thyroglossal duct cysts. Axial (**a**) and sagittal (**b**) contrast-enhanced CT images of the neck demonstrate the close relationship of a thyroglossal duct cyst (non-enhancing cystic structure within the midline neck) with the hyoid bone (arrows). **c** Sagittal T2-weighted MR image of a different patient reveals a fluid signal lesion extending posterior to the hyoid bone (arrow)

**Fig. 3:**
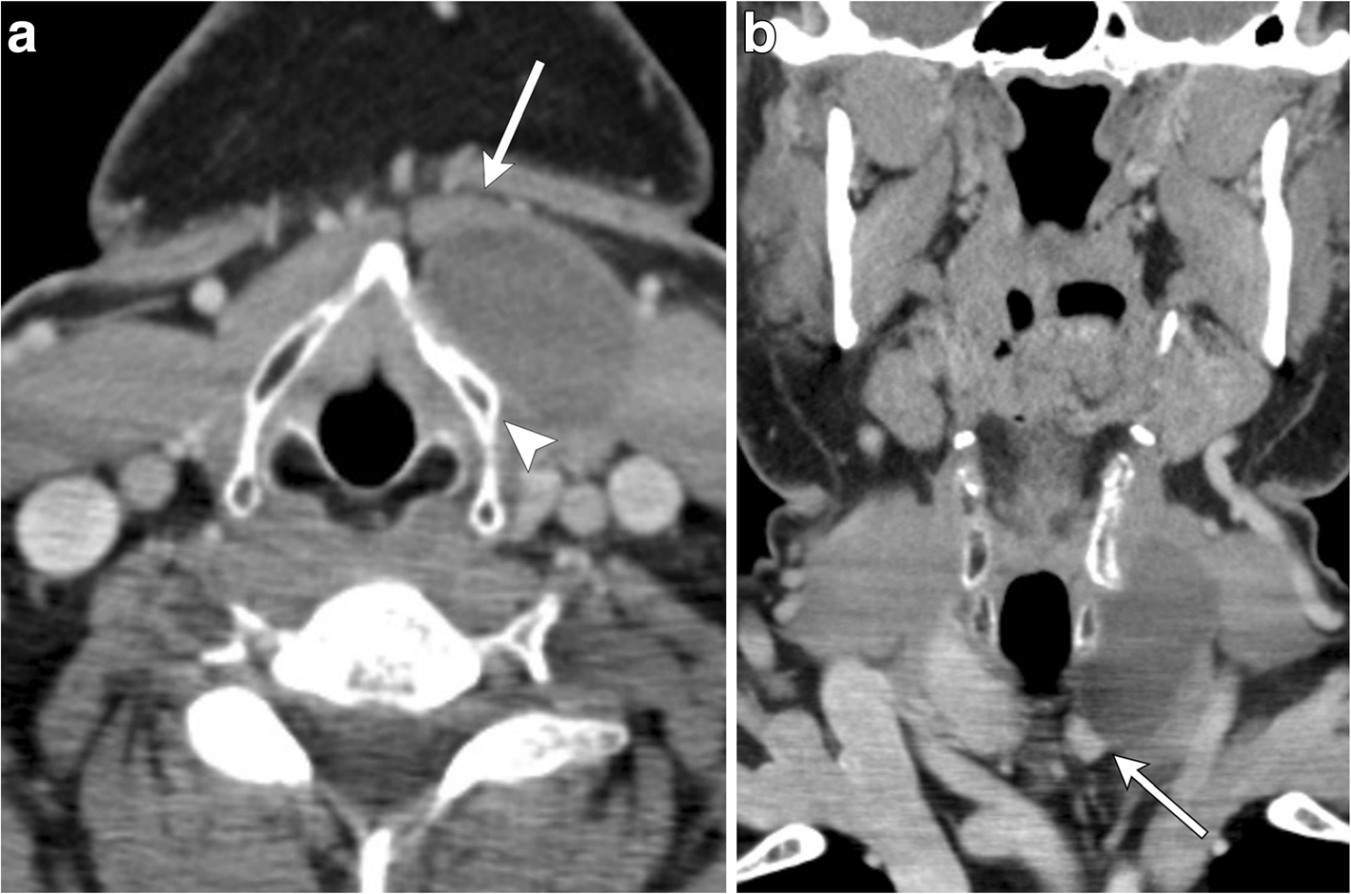
Paramidline thyroglossal duct cyst. **a** Axial contrast-enhanced CT images of the infrahyoid neck demonstrate a well-circumscribed homogenous low attenuation mass located between the thyroid cartilage (arrowhead) and strap muscle (arrow). **b** Coronal contrast-enhanced CT image shows the paramidline location and extension to upper pole of the left thyroid lobe (arrow)

**Fig. 4:**
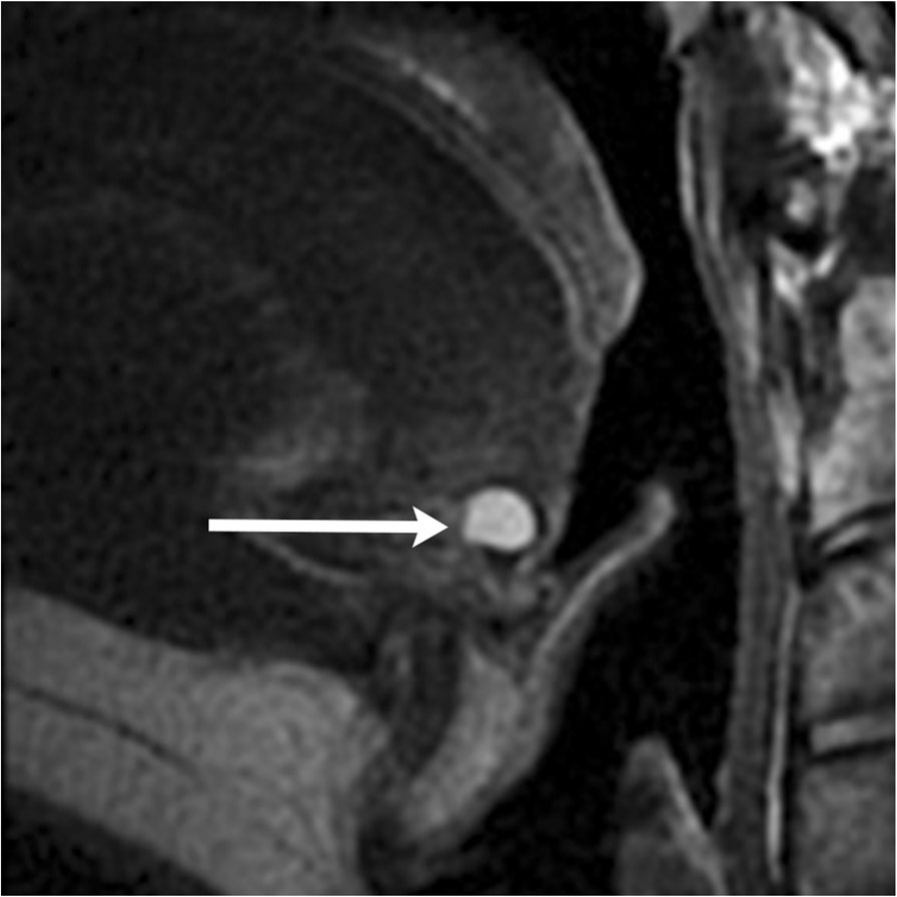
Lingual thyroglossal duct cyst. Sagittal T2-weighted MR image demonstrates a fluid signal lesion at the foramen cecum (arrow)

**Fig. 5:**
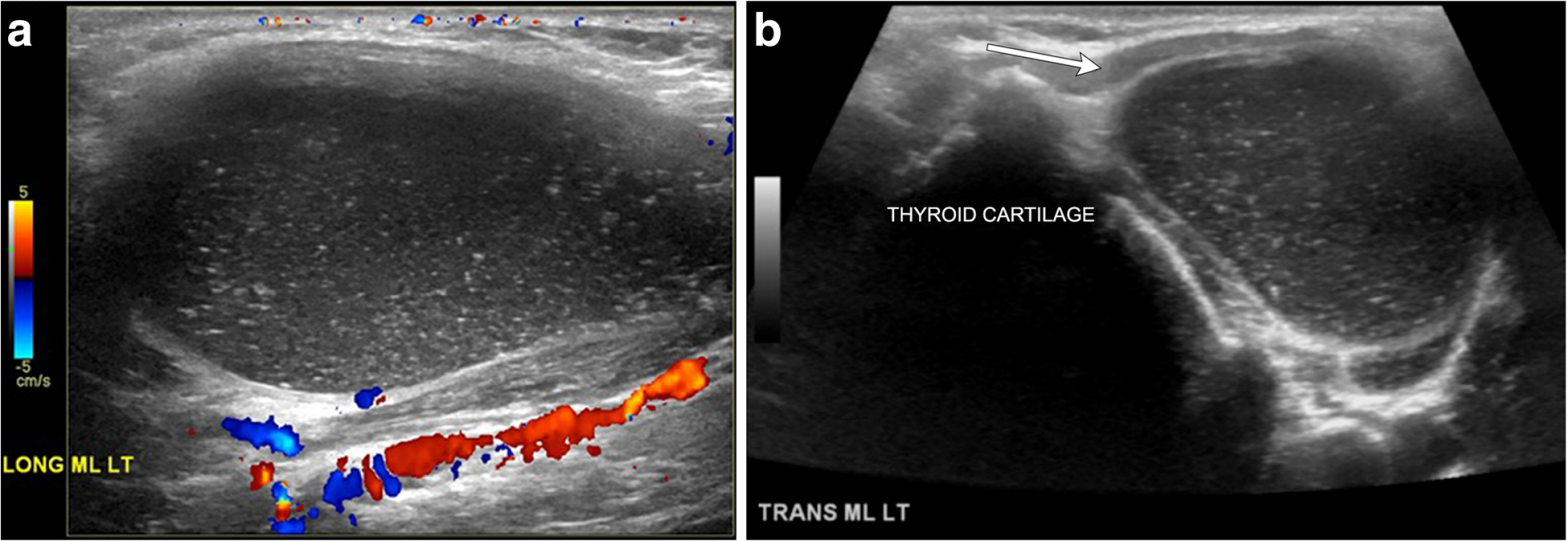
Thyroglossal duct cyst containing debris. **a** Long axis grayscale ultrasound image with color Doppler demonstrates a well-circumscribed hypoechoic structure containing tiny hyperechoic foci compatible with debris. Note the lack of vascularity within the lesion and posterior through transmission. **b** Grayscale ultrasound image illustrates the paramidline location adjacent to the thyroid cartilage and deep to the strap muscle (arrow)

On imaging, suprahyoid thyroglossal duct cysts are generally midline simple or complex cystic structures, while infrahyoid cysts are commonly paramedian in location. CT will show a smooth, thin-walled hypoattenuating mass (Figs. 2 and 3). On MRI, the lesion will be high signal on T2-weighted images, and low to intermediate signal on T1-weighted images, depending on the degree of proteinaceous or hemorrhagic contents (Figs. 2 and 4) [8]. Ultrasound is also sometimes used for evaluation, especially in the pediatric population, which will show a well-circumscribed anechoic to hypoechoic structure with posterior through transmission; there may be some internal debris (Fig. 5) [3].

**Fig. 6:**
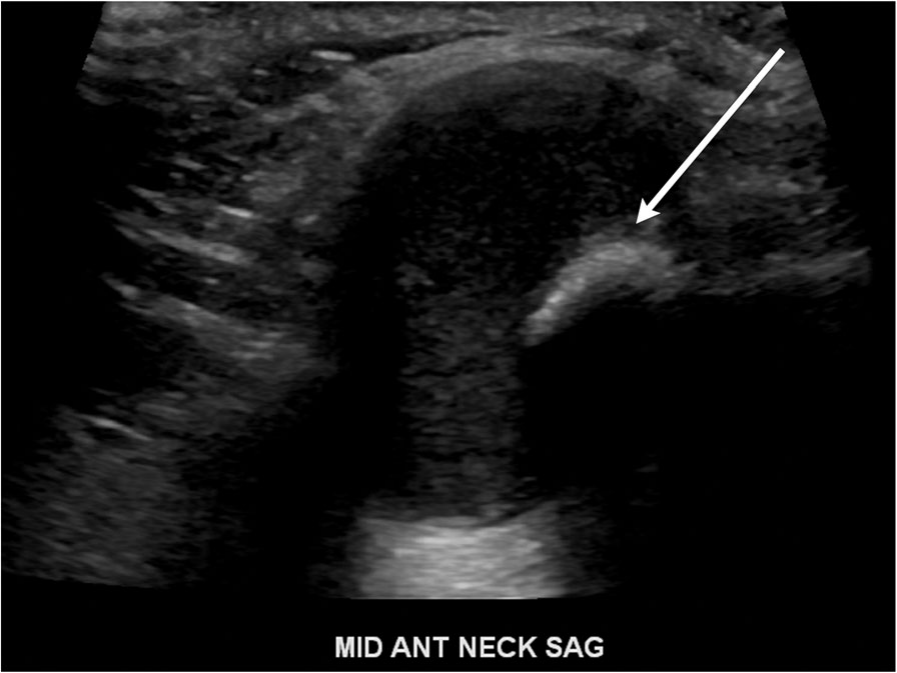
Infrahyoid thyroglossal duct cyst. Long axis grayscale ultrasound image shows a midline cyst in contact with and extending posterior to the hyoid bone (arrow)

**Fig. 7:**
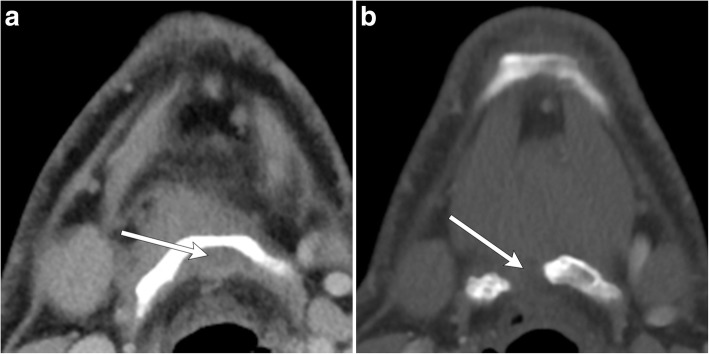
Sistrunk procedure. **a** Axial contrast-enhanced CT image demonstrates a thyroglossal duct cyst wrapping around the hyoid bone (arrow). **b** Post-operative axial contrast-enhanced CT image demonstrates removal of the thyroglossal duct cyst and a partial hyoidectomy (arrow)

During development, the thyroglossal duct wraps inferiorly around the hyoid bone; therefore, a cystic lesion in close association within the hyoid can clue one into the diagnosis (Figs. 2 and 6) [5, 9]. On rare occasions, the duct may become trapped and incorporated into the second and third arch components of the hyoid bone. As such, the Sistrunk resection involves removal of the hyoid body, as well as the entire thyroglossal duct tract and a portion of the tongue base to minimize local recurrence (Fig. 7) [10].

### Infected thyroglossal duct cyst

**Fig. 8:**
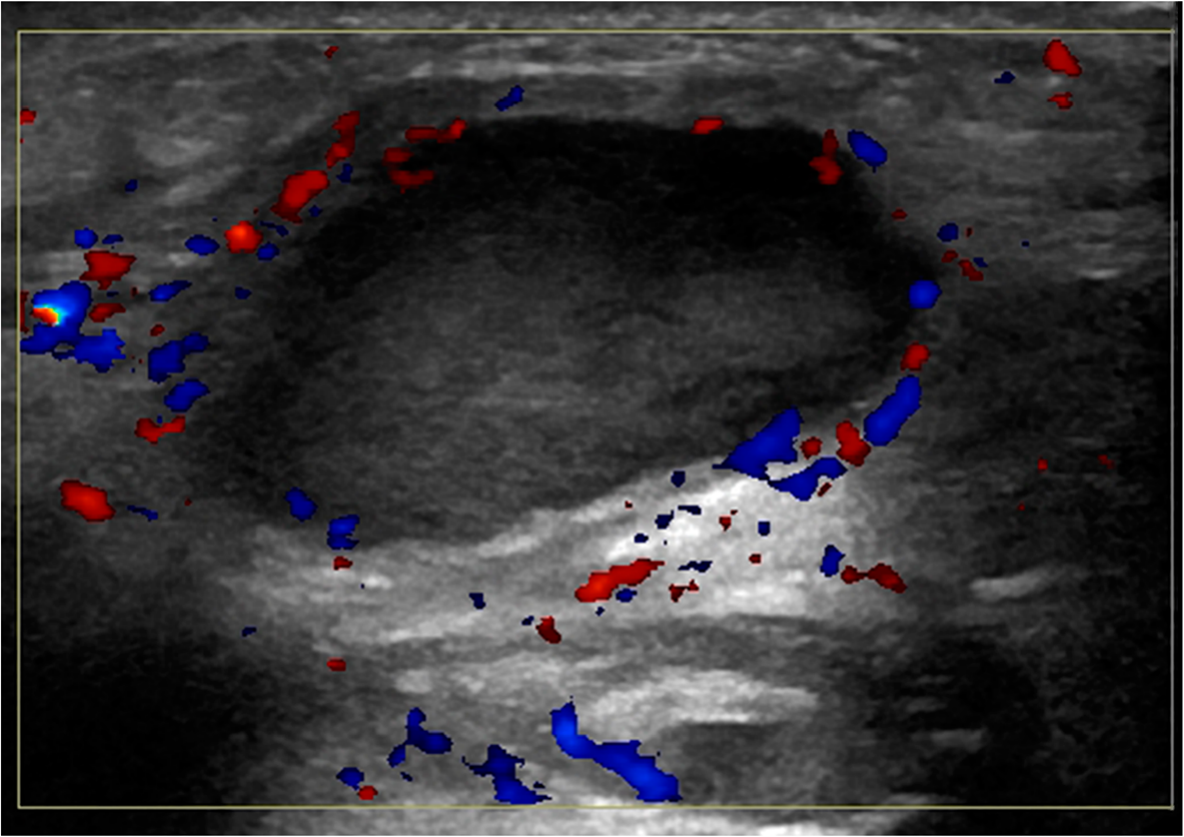
Infected thyroglossal duct cyst. A 23-year-old male presents with several days of fever, as well as a warm anterior neck mass. Long axis ultrasound image with color Doppler reveals a thick-walled hypoechoic structure containing low level echoes with peripheral hyperemia in the paramidline anterior neck

**Fig. 9:**
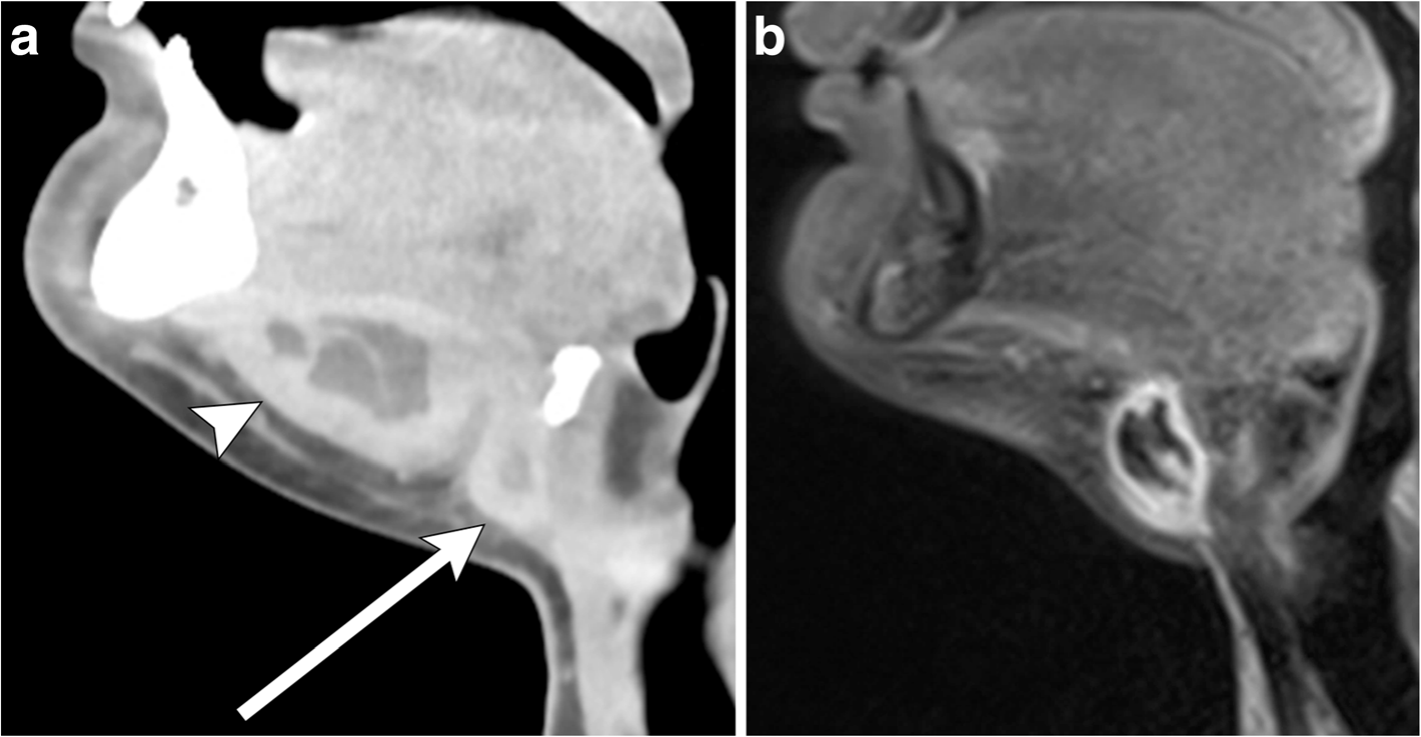
Infected thyroglossal duct cyst with abscess. **a** Sagittal contrast-enhanced CT image of the neck shows a thick-walled cyst adjacent to the hyoid bone (arrow). There is a communicating peripherally enhancing fluid collection along the floor of the mouth, consistent with abscess (arrowhead). Note stranding of the adjacent fat. **b** Sagittal post-contrast T1-weighted MR imaging of the neck demonstrates interval resolution of the abscess and underlying thyroglossal duct cyst with residual inflammatory changes. Note the relative increased thickness of the wall of the infected thyroglossal duct cyst compared to one that is not infected (see Fig. 2)

Some thyroglossal duct cysts may not appear as simple thin-walled unilocular lesions. Presence of internal high attenuation, internal debris, and septations generally correlates with prior infection. In active or recent infection, patients may complain of tenderness at the site of a rapidly growing neck mass. Subsequent imaging reveals a thick-walled cyst with rim enhancement and inflammatory changes of the surrounding subcutaneous tissues **(**Fig. 8**)**. Internal contents may vary, with higher complexity reflecting proteinaceous debris, which may be seen in acute or remote infection [11]. In advanced cases, abscess formation can occur (Fig. 9); these will show restricted diffusion on MR imaging if there is doubt on ultrasound or CT [12]. Fistula formation may develop in severe infections with external cyst rupture or recurrence after resection, although congenital fistula in the newborn have been reported related to complete persistence of the thyroglossal tract after birth (7). Acquired fistulas can be distinguished apart from congenital cases based on later age of presentation (late childhood to early adulthood) and focal irregularity; focal enlargement of the tract on fistulography may represent the site of a ruptured cyst or prior resection [13].

### Malignancy in a thyroglossal duct cyst

Coexisting carcinoma is rare, occurring in less than 1% of patients and usually arises from thyroid remnants entrapped within the cyst during development. Often these carcinomas are incidentally diagnosed on surgical pathology as the initial disease burden may be microscopic with slow growth. Although thyroglossal duct cysts are anomalies commonly occurring in the pediatric population, coexisting carcinoma usually occurs in patients 40 years of age or older [14]. All subtypes of thyroid carcinoma have been described in thyroglossal duct cysts with the exception of medullary carcinoma due to lack of parafollicular cells in the thyroid anlage. The vast majority of cases represent papillary carcinoma, similar to orthotopic thyroid malignancy. Despite the lack of established predisposing factors, radiation therapy is considered a risk factor along with a female predominance.

**Fig. 10:**
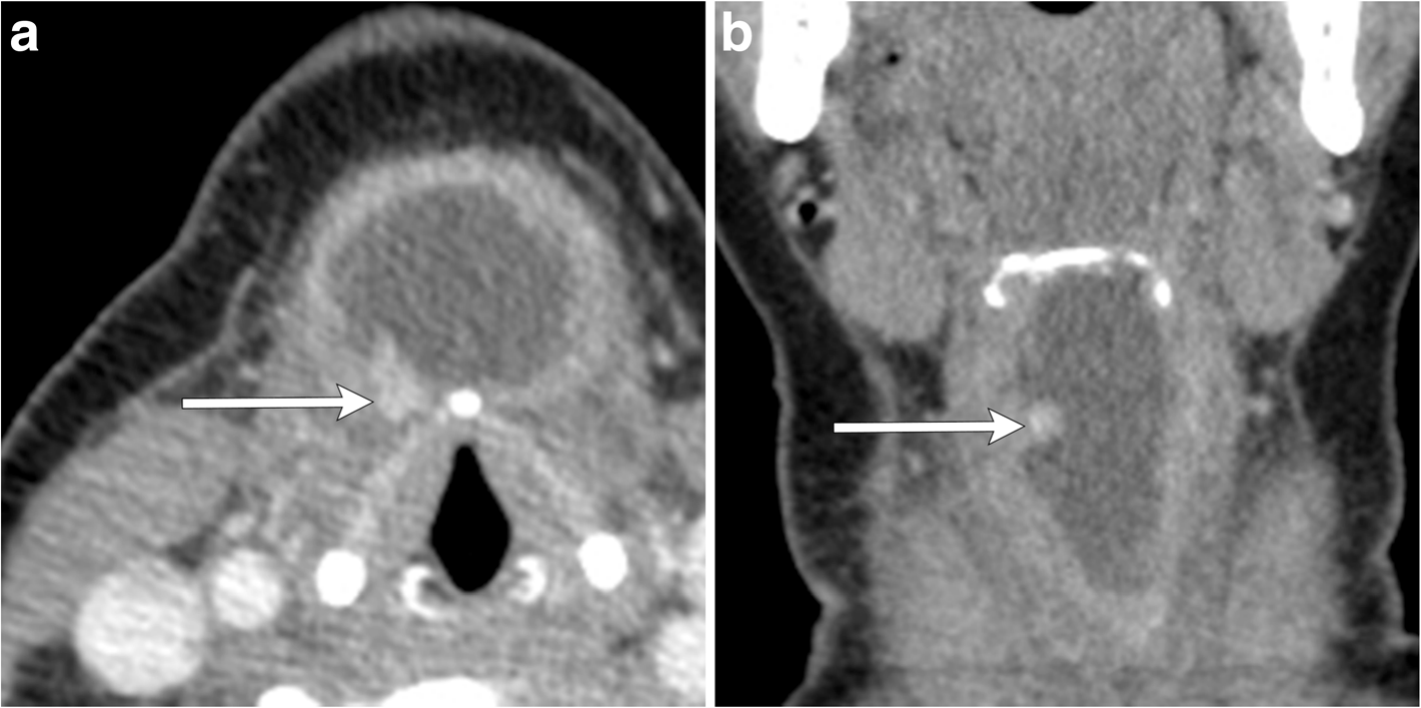
Thyroglossal ductal cyst with malignancy. Axial (**a**) and coronal (**b**) contrast-enhanced CT images of the neck show an irregularly thick-walled thyroglossal duct cyst with enhancing mural nodule (arrows). Resection revealed papillary carcinoma

**Fig. 11:**
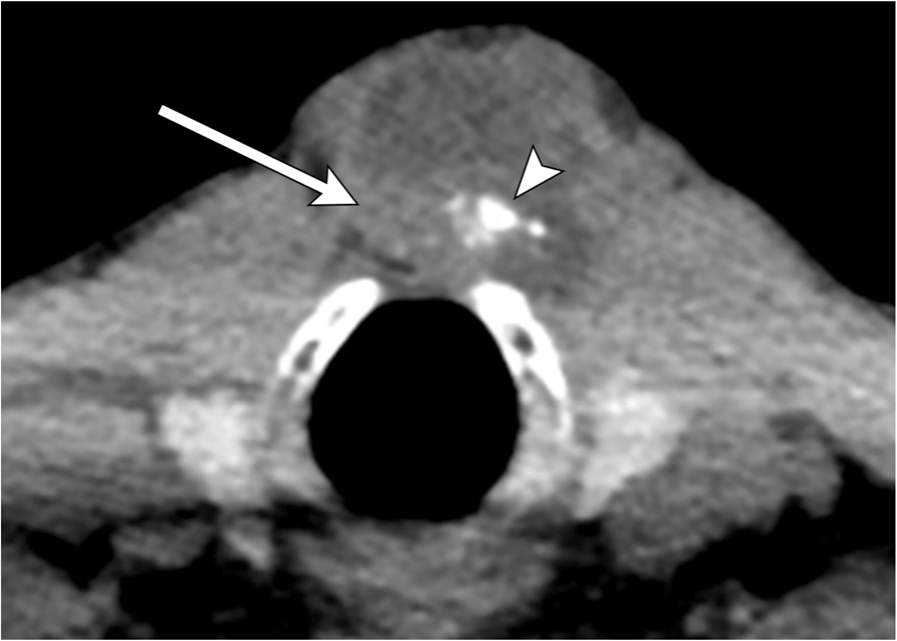
Thyroglossal duct cyst with malignancy. Axial CT image demonstrates a thyroglossal duct cyst, which is predominantly cystic, but also has soft tissue attenuation (arrow) and calcifications (arrowhead) at the posterior aspect. Resection revealed papillary carcinoma

**Fig. 12:**
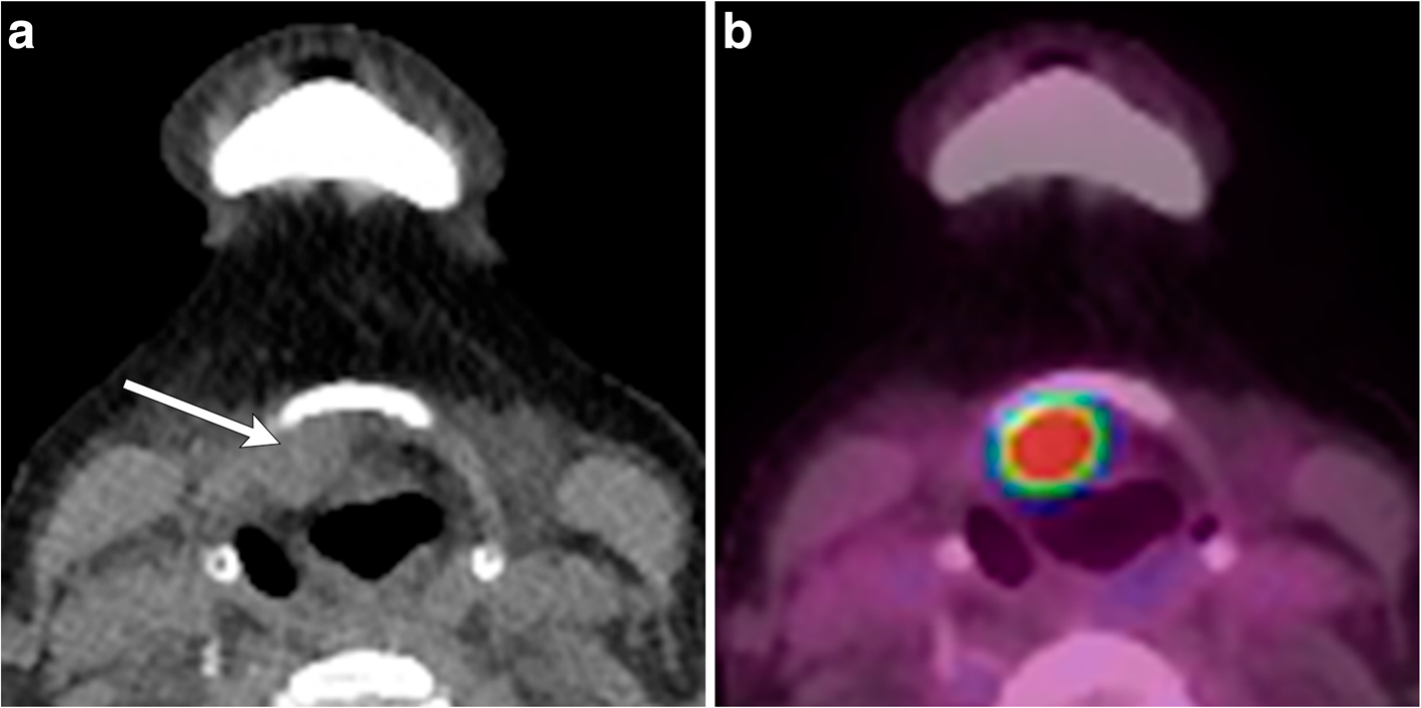
Malignancy of thyroglossal duct remnant. **a** Axial non-contrast CT image demonstrates a soft tissue mass adjacent to the posterior margin of the hyoid bone (arrow). **b** Axial fused PET/CT image demonstrates FDG avidity in this lesion. Pathology proven papillary carcinoma

On imaging, commonly described features include enhancing wall nodularity and calcifications within the thyroglossal duct cyst (Figs. 10 and 11). Calcification is not usually appreciated on MR imaging, requiring a supplementary CT examination [4, 6]. Calcifications are a more specific indicator of malignancy than solid components, as the latter can also be seen in inflammatory processes. Rarely, a purely solid midline or paramidline lesion may be malignant and should be considered when there is associated central FDG avidity or the presence of suspected regional metastatic lymphadenopathy (Fig. 12) [14]. Fine needle aspiration cytology may be obtained of suspicious solid components to confirm the diagnosis [15].

### Mimics of thyroglossal duct cysts

There are many mimics of thyroglossal duct cysts, and it is important to recognize these as each has different clinical implications. Close attention to the age of presentation, location of the lesion, association with surrounding structures, and internal architecture can clue one into the correct diagnosis (Table 1).

**Table 1:** Distinguishing imaging features of cystic lesions in the anterior neck

	Differentiating features of cystic lesions
Thyroglossal duct cyst	Close association with posterior aspect of hyoid
	Midline in suprahyoid neck, may be paramidline in infrahyoid neck
Branchial (second) cleft cyst	Located along anteromedial border of the sternocleidomastoid muscle, lateral to the carotid space, and at the posterior margin of the submandibular gland
	Beak sign may be present (curved rim of the lesion extending between the internal and external carotid arteries)
Dermoid/epidermoid	Located in subcutaneous tissues, superficial to strap musculature, typically near suprasternal notch
	Presence of fat or calcification
Laryngocele	May be primarily air-filled or with air-fluid levels due to airway communication
	Involvement of laryngeal ventricle
Thymic cyst	Close association with the carotid sheath, sometimes splaying the carotid artery and jugular vein
	Dumbbell or bilobed appearance can be seen with extension into the anterior mediastinum
Necrotic metastases	Multiple, growing masses
	Irregular solid and cystic components
	Calcifications suggestive of papillary thyroid carcinoma
Lymphatic malformation	Trans-spatial lesion
	Fluid-fluid levels from recent hemorrhage

## Branchial cleft cysts

**Fig. 13:**
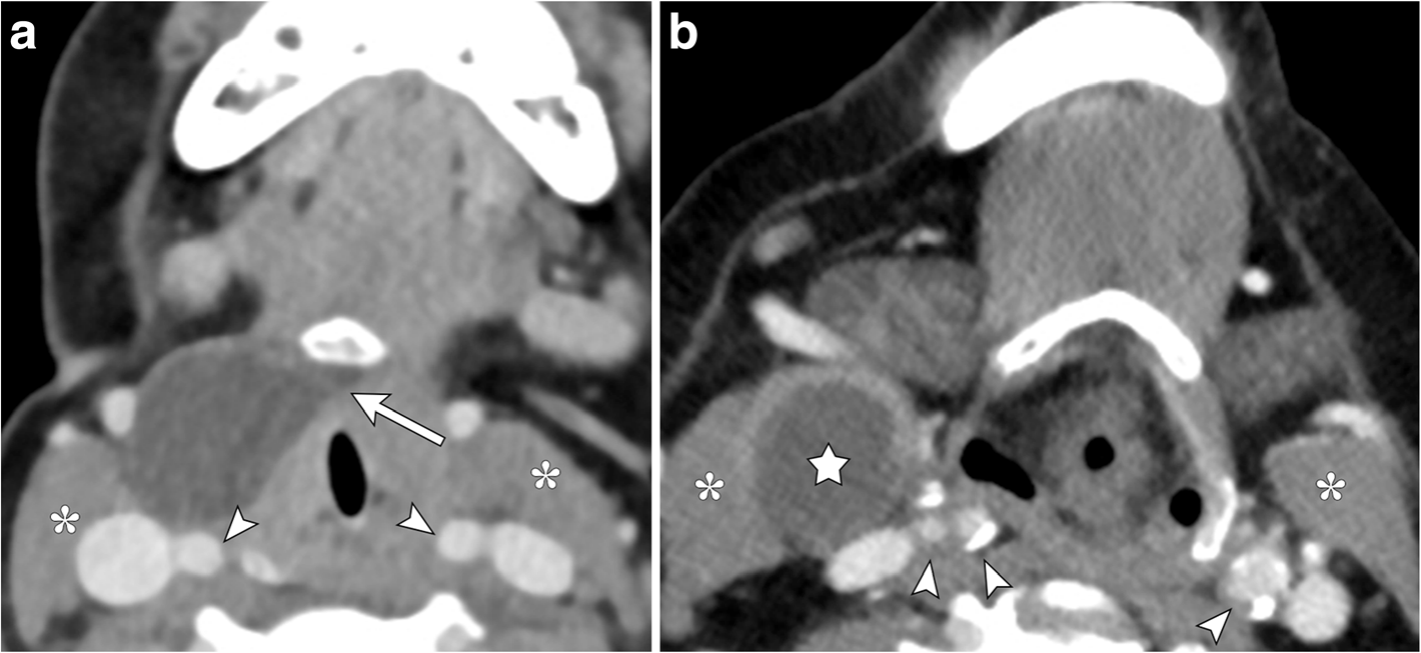
Thyroglossal duct cyst and branchial cleft cyst. **a** Axial contrast-enhanced CT image shows a pathology proven thyroglossal duct cyst located anterior to the carotid vessels (arrowheads) and anteromedially to the sternocleidomastoid muscle (asterisks) with a tail-like extension medially to the hyoid bone (arrow). **b** Axial contrast-enhanced CT image demonstrates a second branchial cleft cyst (star) in a similar location within the right neck without hyoid extension

Branchial cleft cysts are congenital lesions which usually present after upper respiratory infection and most commonly arise from the second branchial cleft. These typically are located laterally in the anterior neck, adjacent to the anterior surface of the sternocleidomastoid muscle and lateral to the carotid space and posterior to the submandibular gland, often associated with a sinus tract or fistula. A lateral suprahyoid thyroglossal duct cyst can be distinguished by the presence of a medial tail-like component extending into the hyoid bone (Fig. 13). A second branchial cleft cyst may occasionally demonstrate a beak sign; however, the curved rim of the lesion will tend to extend between the internal and external carotid arteries [16, 17].

## Dermoid and epidermoid cysts

**Fig. 14:**
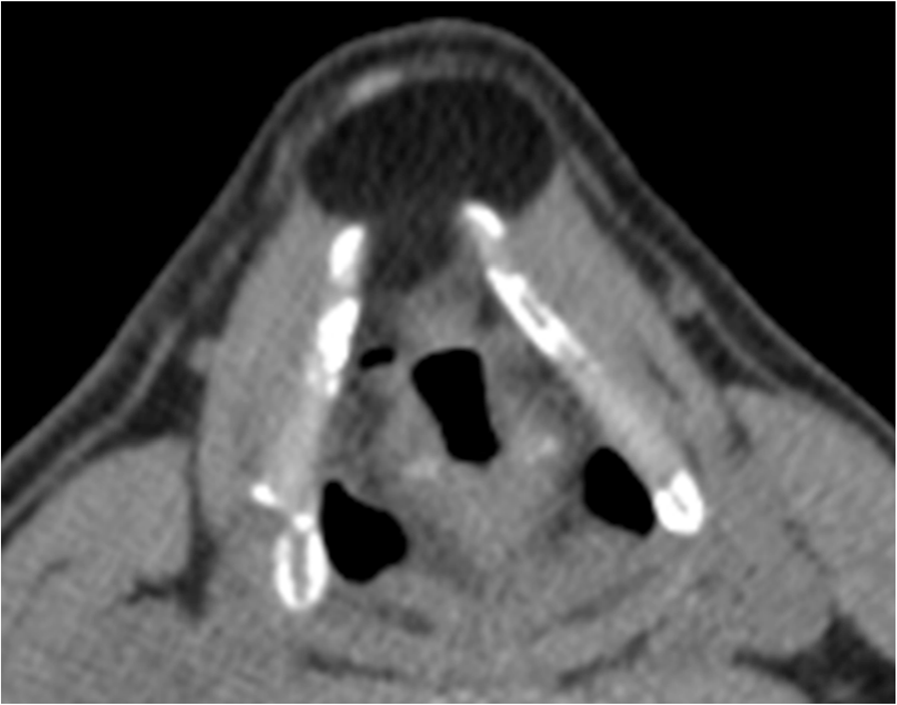
Dermoid. Axial non-contrast CT image illustrates a neck mass located anterior to the thyroid cartilage and superficial to the strap muscles. The lesion demonstrates the same attenuation as adjacent subcutaneous fat

Dermoid and epidermoid cysts represent a part of the spectrum of congenital and acquired cystic malformations sharing the common characteristic of a squamous epithelial lining. Dermoid cysts will be differentiated by internal calcific and fat content (Fig. 14). Epidermoid cysts will show diffusion restriction [8]. Dermoid and epidermoid cysts arise from dermal elements of the first and second branchial arches, and therefore are located at base of tongue and superficially within the subcutaneous tissues of the anterior neck. In contrast, thyroglossal duct cysts are classically in a deeper location, embedded within strap musculature and in close proximity to the hyoid bone [18].

## Laryngocele

**Fig. 15:**
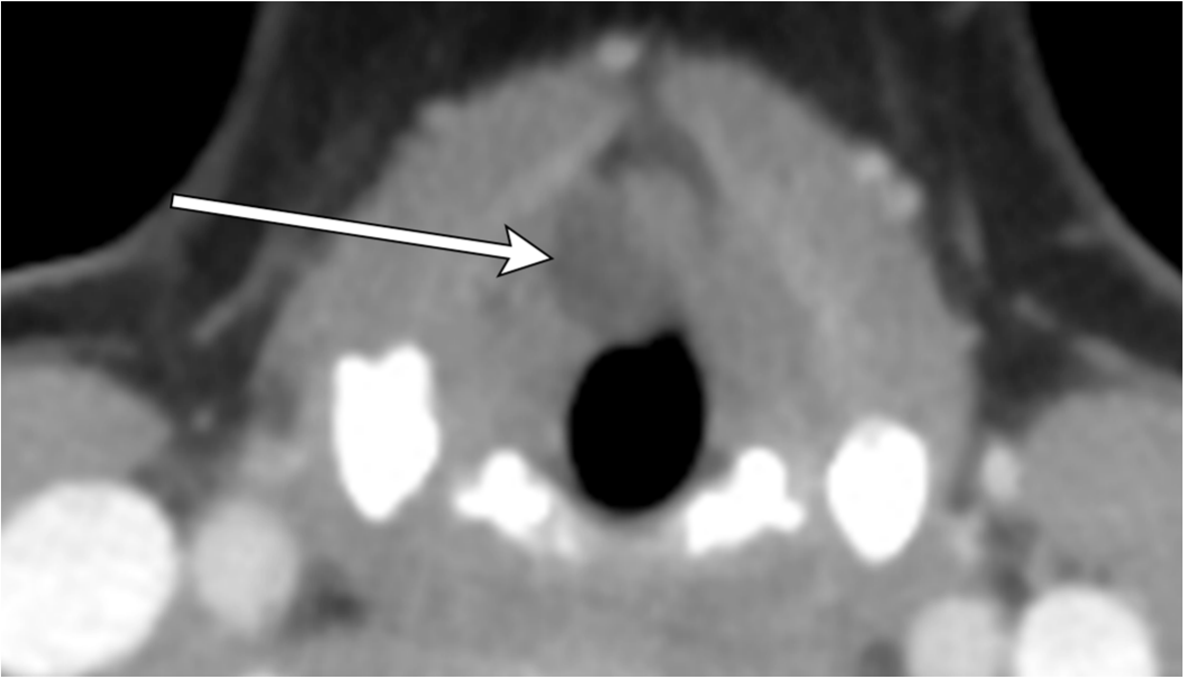
Internal laryngocele. Axial contrast-enhanced CT image shows a non-enhancing fluid attenuating structure in right paraglottic region with mass effect on false vocal cord (arrow)

Saccular cysts and laryngoceles are considered congenital dilatations of the saccule of the laryngeal ventricle in the supraglottic larynx. They are classified as internal, external, or mixed based on relationship of saccular dilatation to the thyrohyoid membrane. Both these and thyroglossal duct cysts can extend through the thyrohyoid membrane; however, thyroglossal duct cysts do not involve the laryngeal ventricle. Additionally, laryngoceles may be fluid-filled, or have air-fluid levels due to airway communication (Fig. 15) [1, 16].

## Thymic cyst

**Fig. 16:**
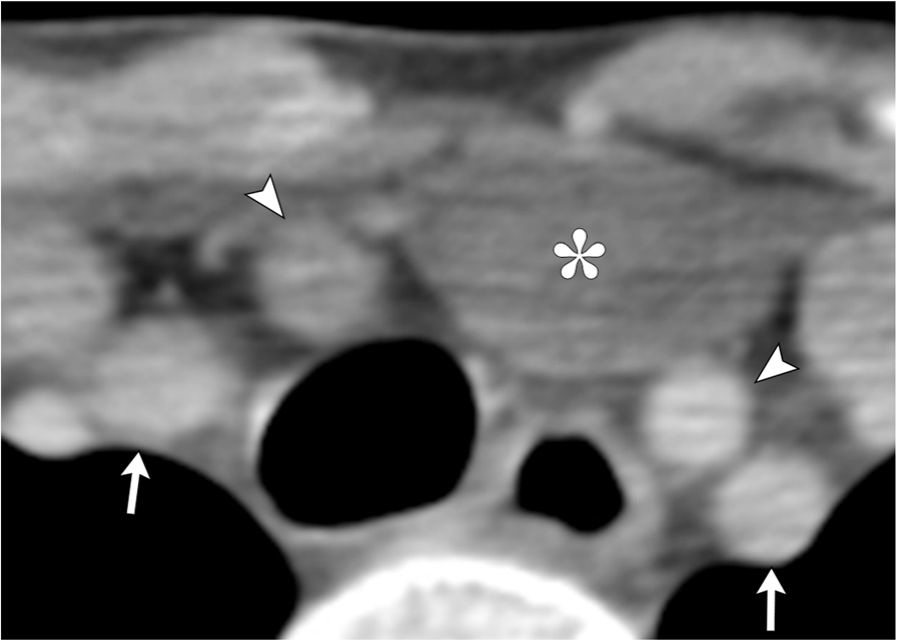
Thymic Cyst. Axial contrast-enhanced CT image demonstrates a cystic mass (asterisk) in the left neck base between the right and left common carotid arteries (arrowhead), extending into the superior mediastinum. Bilateral subclavian arteries are also visualized (arrows)

Thymic cysts are rare cystic lesions arising from the persistent thymopharyngeal duct, which extends from the pyriform sinus to anterior mediastinum. They are located in the lateral infrahyoid neck, predominantly occurring on the left side (Fig. 16). They can be distinguished based on the close association with the carotid sheath, sometimes splaying the carotid artery and jugular vein; the classic dumbbell or bilobed appearance can be seen with extension into the anterior mediastinum [11].

## Necrotic lymph nodes

**Fig. 17:**
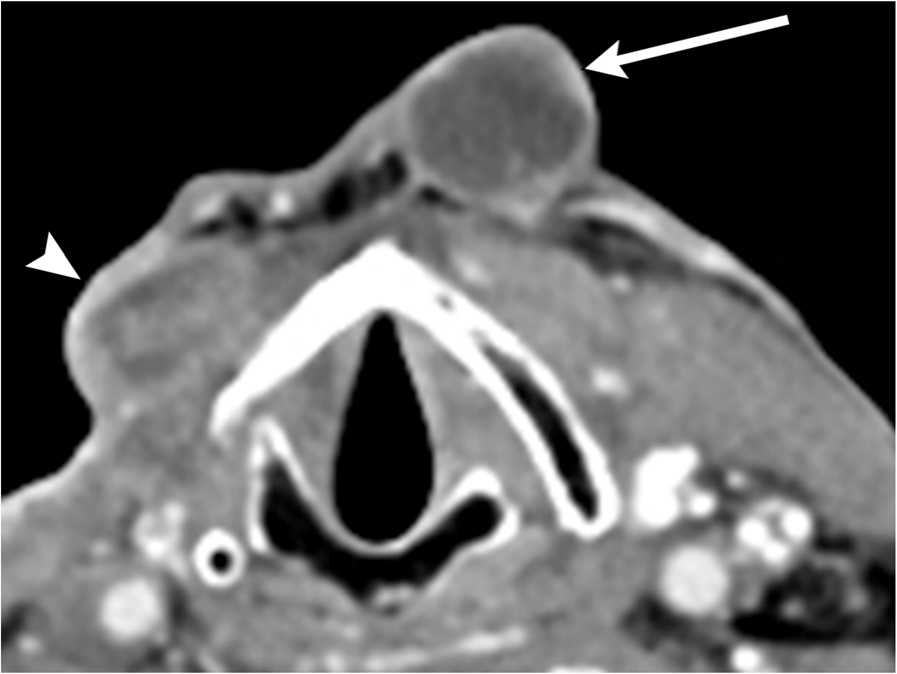
Necrotic metastatic lymphadenopathy (primary later found to be tongue base squamous cell carcinoma). Axial contrast-enhanced CT image illustrates an irregularly thick-walled cystic mass anterior to the left thyroid cartilage (arrow). Careful search in other areas of the neck demonstrate another similar appearing cystic lymph node adjacent to the right thyroid cartilage (arrowhead)

Nodal metastases of head and neck neoplasms account for about 80% of cystic neck masses in adults over 40 years of age [16]. Most commonly arising from papillary thyroid carcinoma or head and neck squamous cell carcinoma, necrotic metastatic lymph nodes are important to identify as they significantly alter prognosis and management. Hallmark imaging findings include multiple, enlarging, round masses with central cystic necrosis, eccentric solid component(s), and disruption of the usual fatty hilar architecture (Fig. 17). Multiplicity and irregular central cystic changes can be used to distinguish from congenital cystic neck anomalies. Punctate calcifications may suggest metastases originating from papillary thyroid carcinoma. Hypermetabolism can be confirmed on PET imaging. Additionally, advanced MR imaging with diffusion-weighted or dynamic contrast-enhanced sequences have been shown to differentiate benign from malignant nodes based on low ADC value or enhancement kinetics, respectively [19, 20]. Further discussion of these techniques is beyond the scope of this article.

## Lymphatic malformation

**Fig. 18:**
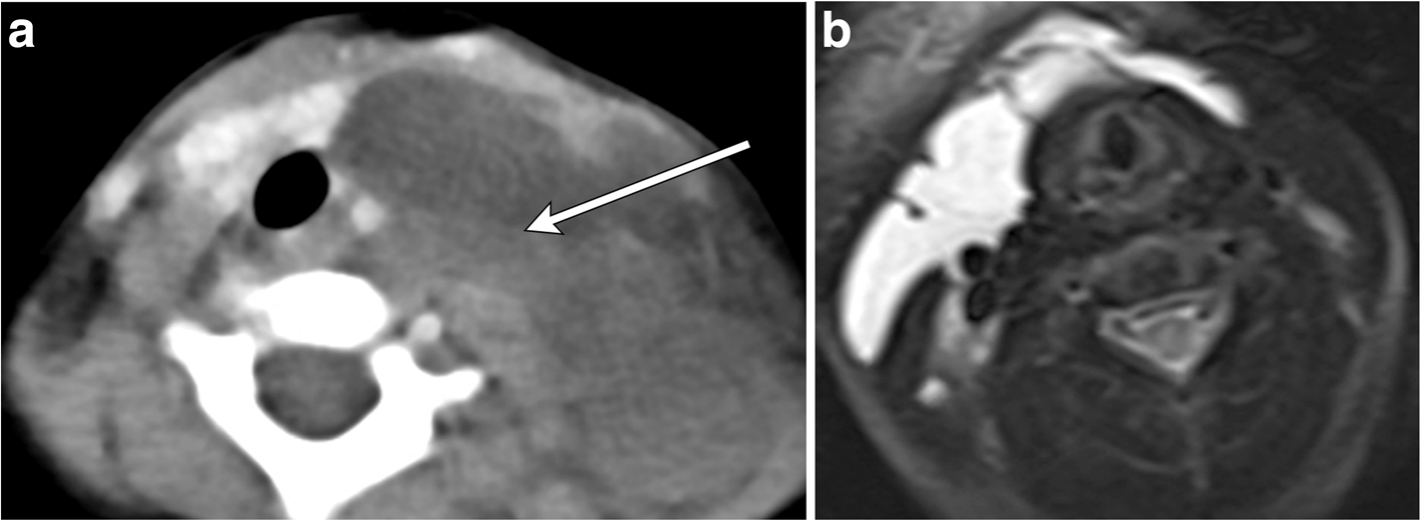
Lymphangioma. **a** Axial contrast-enhanced CT image demonstrates a non-enhancing multilocular, trans-spatial fluid-attenuation mass centered in the left neck base with mass effect and rightward displacement of trachea and esophagus. Note the fluid-fluid levels indicative of prior hemorrhage (arrow), distinguishing this lesion from a thyroglossal duct cyst. **b** Axial T2-weighted MR image in a different patient demonstrates a trans-spatial multiseptated cystic mass extending from the right lateral infrahyoid neck across the anterior aspect of the thyroid cartilage

Arising from sequestration of variably sized embryonic lymphatic channels, lymphatic malformations can occur in almost any location with a predilection in the head and neck, along the jugular vessels. Classified based on size of lymphatic cavities, these infiltrative lesions are most commonly seen in children by age two with commensurate growth and compression of surrounding neck structures leading to clinical presentation. Lobulated, multiseptated cystic appearance may appear similar to an infected thyroglossal duct cyst; however, the presence of fluid-fluid levels from recent hemorrhage, trans-spatial growth pattern, and presence of traversing venous vessels are key distinguishing features of lymphatic malformations (Fig. 18) [21]. In conjunction with conventional MR, dynamic contrast-enhanced MR imaging can be performed to distinguish low flow lesions such as lymphatic malformations from high-flow lesions such as arteriovenous malformation or fistula. The presence of late peak enhancement, dilated venous spaces, and lack of flow voids are suggestive of low-flow malformations, while early peak enhancement and presence of flow voids are indicative of high-flow malformations [22].

### Ectopic (lingual) thyroid

**Fig. 19:**
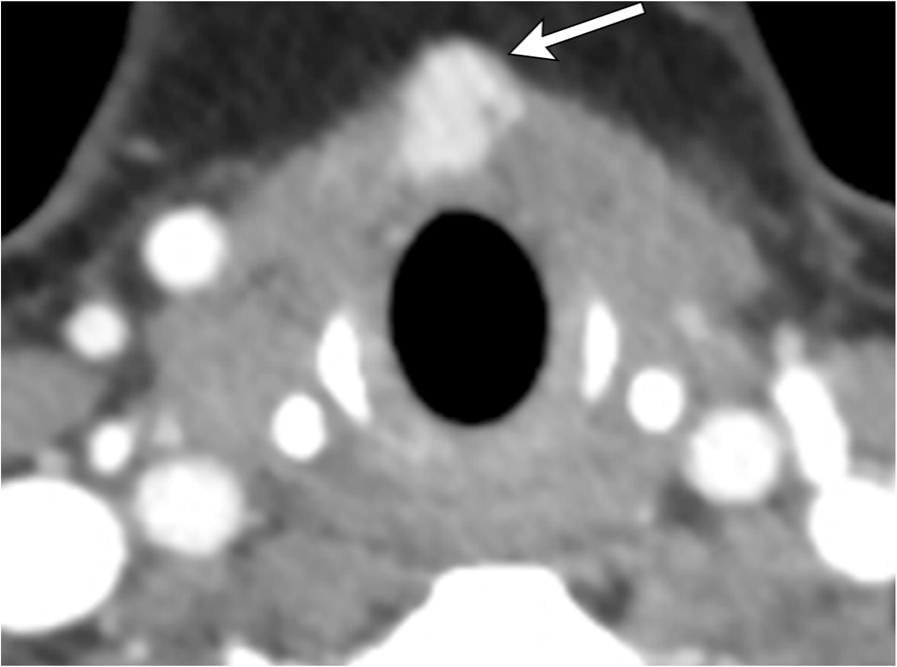
Ectopic thyroid tissue. Axial contrast-enhanced CT image demonstrates a focal high attenuating soft tissue mass in the midline anterior neck along the anterior margin of the thyroid cartilage (arrow). Invaginated by strap musculature, this mass resembles the thyroid gland in attenuation and occurs in expected course of the thyroglossal duct tract

As eluded to earlier, thyroid tissue remnants can be found anywhere along the midline tract of the thyroglossal duct, extending from the foramen cecum to the thyroid cartilage (Fig. 19). Ectopic thyroid tissue depositing lateral to the expected midline course is rare [1, 23]. About 90% of reported cases occur at the base of the tongue, and therefore, are called lingual thyroid [18]. Usually asymptomatic and incidentally discovered, a lingual thyroid may come to clinical attention with development of dysphagia and stridor, most commonly in children [24].

**Fig. 20:**
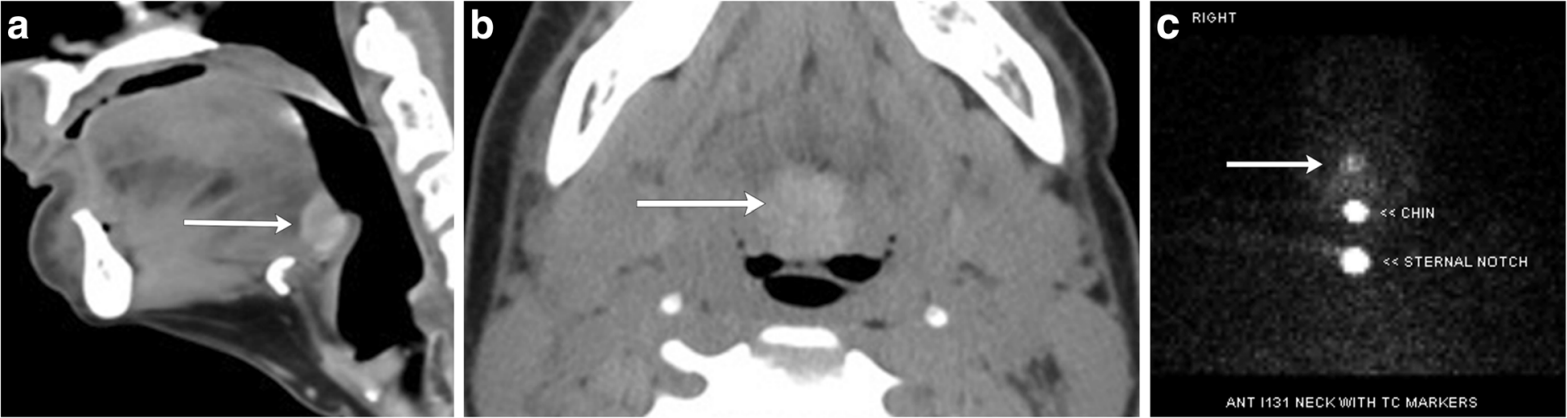
Lingual thyroid. **a** Sagittal contrast-enhanced CT image of the neck demonstrates a homogenously enhancing lingual thyroid at the base of the tongue encroaching on the valleculae (arrow). **b** Axial non-contrast CT shows the inherently high attenuating midline mass at the base of the tongue (arrow). **c** I-123 thyroid scan confirms the diagnosis, expected iodine uptake of the lingual thyroid (arrow)

A lingual thyroid demonstrates the same imaging characteristics as normal thyroid tissue: high attenuation structure relative to adjacent muscle on CT with diffuse enhancement and high signal on T1-weighted images with variable enhancement on MRI [1, 23]. As expected, lesions of normal thyroid tissue such as nodules can also be seen in lingual thyroid and serve as key diagnostic clues [25]. It is important to closely inspect the thyroid bed in cases of lingual thyroid when surgery is planned for removal, as orthotopic tissue is absent in about 70–80% of cases; a patient with little or no thyroid tissue in the expected bed warrants supplemental thyroid hormone after surgery [26]. Radionuclide technetium 99 m-pertechnetate or radioiodine imaging (utilizing I^123^ or I^131^) can confirm the diagnosis of ectopic thyroid tissue (Fig. 20) [27].

## Conclusion

Accurate assessment of anterior neck masses requires awareness of the embryological path of thyroid development for recognition of thyroglossal duct anomalies, variants, and complications. Knowledge of differentiating features of other cystic neck masses is important due to different clinical implications.
